# Phylogeny and Phylogeography of Rhizobial Symbionts Nodulating Legumes of the Tribe Genisteae

**DOI:** 10.3390/genes9030163

**Published:** 2018-03-14

**Authors:** Tomasz Stępkowski, Joanna Banasiewicz, Camille E. Granada, Mitchell Andrews, Luciane M. P. Passaglia

**Affiliations:** 1 Autonomous Department of Microbial Biology, Faculty of Agriculture and Biology, Warsaw University of Life Sciences (SGGW), Nowoursynowska 159, 02-776 Warsaw, Poland; banasiewicz.joanna0@gmail.com; 2 Universidade do Vale do Taquari—UNIVATES, Rua Avelino Tallini, 171, 95900-000 Lajeado, RS, Brazil; cegranada@univates.br; 3 Faculty of Agriculture and Life Sciences, Lincoln University, P.O. Box 84, Lincoln 7647, New Zealand; mitchell.andrews@lincoln.ac.nz; 4 Departamento de Genética, Instituto de Biociências, Universidade Federal do Rio Grande do Sul. Av. Bento Gonçalves, 9500, Caixa Postal 15.053, 91501-970 Porto Alegre, RS, Brazil; luciane.passaglia@ufrgs.br

**Keywords:** *Bradyrhizobium*, Genisteae, *Microvirga*, *nifD*, *nodA*, phylogeography

## Abstract

The legume tribe Genisteae comprises 618, predominantly temperate species, showing an amphi-Atlantic distribution that was caused by several long-distance dispersal events. Seven out of the 16 authenticated rhizobial genera can nodulate particular Genisteae species. *Bradyrhizobium* predominates among rhizobia nodulating Genisteae legumes. *Bradyrhizobium* strains that infect Genisteae species belong to both the *Bradyrhizobium japonicum* and *Bradyrhizobium elkanii* superclades. In symbiotic gene phylogenies, Genisteae bradyrhizobia are scattered among several distinct clades, comprising strains that originate from phylogenetically distant legumes. This indicates that the capacity for nodulation of Genisteae spp. has evolved independently in various symbiotic gene clades, and that it has not been a long-multi-step process. The exception is *Bradyrhizobium* Clade II, which unlike other clades comprises strains that are specialized in nodulation of Genisteae, but also *Loteae* spp. Presumably, Clade II represents an example of long-lasting co-evolution of bradyrhizobial symbionts with their legume hosts.

## 1. Introduction: Origin and Differentiation of the Tribe Genisteae

The Genisteae is one of the largest tribes within the legume family (*Fabaceae*) with ca. 618 species within 25 genera [1]. Most genera within the Genisteae show a preference for a temperate climate although several lupin (*Lupinus* spp.) species inhabit tropical areas in eastern Brazil [2,3,4]. These predominantly woody legumes thrive in a wide range of habitats, including coastal dunes, scrubland, sagebrush steppes, grasslands, mountain woodlands, and meadows. Genisteae (especially lupins) inhabit areas across a wide altitudinal range, extending from sea level to the upper elevation limit for plant growth, i.e., ca. 5,000 m [5]. Almost all of the Genisteae species tested can fix atmospheric nitrogen via symbiotic bacteria (general term ‘rhizobia’) in root nodules, and this gives them an advantage in low N soils if other factors are favourable for growth [6,7,8].

It has been estimated that the separation of the Genisteae and Crotalarieae took place in the Eocene ca. 41 million of years ago [9]. Evidence indicates that the formation of the two basal lineages, which later evolved into the extant genera *Dichilus*-*Melolobium*-*Polhillia* and *Argyrolobium,* occurred in southern Africa. Later, this tribe dispersed northward reaching the Mediterranean, where two other genera, *Lupinus* and *Adenocarpus* appeared. These events were followed by the emergence of the Cytisinae (nine genera) and Genistinae (eight genera) as predominant Genisteae groups in the Mediterranean [1,2,10]. Given that the time of separation of the genus *Lupinus* and the *Cytisinae-Genistinae* genera has been estimated at ca. 16 million years ago, the process of differentiation into the extant Genisteae genera may have been initiated in the Oligocene, albeit not later than in the early Miocene [11,12]. The environmental changes that are associated with the global climate cooling and growing aridification from the end of the Eocene may have played pivotal roles in the Genisteae speciation process [13,14]. Similarly, the mountain uplifts in the western parts of North and South America triggered replicate radiations in the genus *Lupinus*, resulting in the appearance of ca. 200 species with a great variety of morphological forms [12,15].

**Figure 1:**
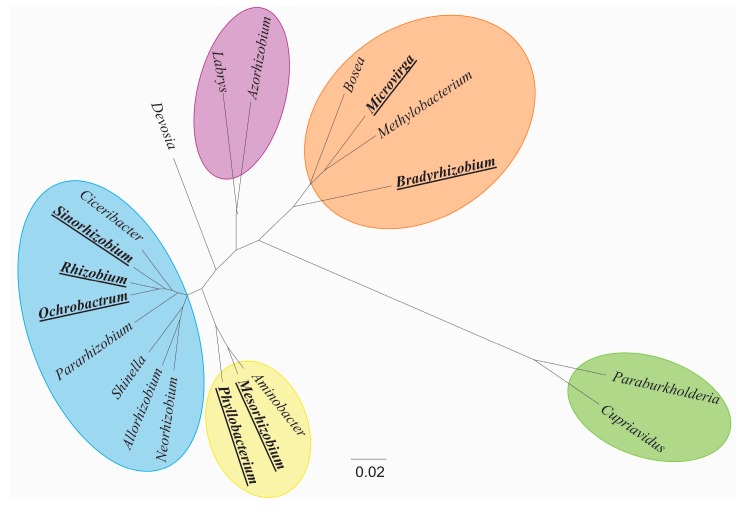
A Bayesian posterior probability consensus tree based on 1408 bps of 16S rRNA derived from 16 rhizobial and 4 related genera (the genera nodulating Genisteae are in bold case-underlined). The strains used for this construction and the 16S rRNA Genbank accession numbers were: *Rhizobium leguminosarum* ATCC 10004 (U29386), *Sinorhizobium* *fredii* ATCC 35423 (X67231), *Allorhizobium undicola ATCC 700741* (Y17047), *Pararhizobium capsulatum* ATCC 43294 (X73042), *Neorhizobium* *galegae* ATCC 43677 (D11343), *Shinella zoogloeoides* ATCC 19623 (AB238789), *Ciceribacter lividus* MSSRFBL1 (NR 135717), *Mesorhizobium loti* ATCC 700743 (X67229), *Aminobacter anthyllidis* STM 4645(FR869633), *Phyllobacterium brassicacearum* LMG 22836 (AY785319), *Ochrobactrum lupini* LMG 22726 (AY457038), *Methylobacterium marchantiae* DSM 21328 (FJ157976), *Bradyrhizobium lupini* USDA 3051 (KM114861), *Bosea lupini* LMG 26383 (FR774992), *Azorhizobium oxalatiphilum* DSM 18749 (FR799325), *Labrys okinawensis* DSM 18385 (AB236169), *Devosia honganensis* ACCC 19737 (KP339871), *Paraburkholderia caribensis* CCUG 42847 (Y17009), *Cupriavidus alkaliphilus* LMG 26294 (HQ438078), and *Microvirga lupini* LMG 26460 (EF191408). The 16S rRNA sequences were aligned in MUSCLE [16] and implemented in MEGA 6.0 [17]. The Bayesian analyses were performed using BEAST 1.7 software [18]. The model of nucleotide evolution used in all of the analyses was GTR + I + G, as selected by the jModel Test software [19]. The Yule process was selected as a tree prior to Bayesian analysis, 10,000,000 generations were performed and the tree was visualized and edited using FigTree version 1.3.1 software [20].

The amphi-Atlantic distribution of Genisteae species points to dispersal events having occurred across the Atlantic Ocean into the areas now comprising a distinct *Fabaceae* flora. The thesis of this review is that the complex evolutionary history of the Genisteae tribe is reflected by the diversity of rhizobium symbionts, with which it interacts. Indeed, seven out of the 16 authenticated rhizobial genera can nodulate particular Genisteae species (Figure 1). Thus, the objective of this work is to present a comprehensive phylogenetic and phylogeographic data concerning the rhizobia that nodulate this legume tribe.

## 2. The Genus *Bradyrhizobium* as a Predominant Group Infecting Genisteae Legumes 

*Bradyrhizobium* strains were isolated from nodules of 30 out of the 33 lupin species listed in Table 1. *Bradyrhizobium* also occured in root nodules of 36 out of the 44 species of the remaining Genisteae genera (see Table 1). This indicates that *Bradyrhizobium* is a dominant rhizobium lineage nodulating this legume tribe although it is acknowledged that rhizobial symbionts have been characterized in only a fraction of Genisteae species [6,21], and nothing is known about rhizobial symbionts of 14, mostly monotypic genera.

The predominance of *Bradyrhizobium* strains among the rhizobia nodulating Genisteae spp. could be due to different factors, including their symbiotic potential that is responsible for their nodulation of a wide spectrum of *Fabaceae* spp. and their adaptability to various edaphic and climatic conditions, resulting in this genus having a broad geographical range [21,97]. Bradyrhizobia are common in soils in places where legumes are absent, which may explain the loss of nodulating ability by portions of rhizobial communities [98,99,100]. Indeed, *Bradyrhizobium* communities comprise both nodulating and non-nodulating bacteria, and the latter may even lack the capacity to fix nitrogen [101]. In phylogenetic trees, these non-nodulating, non-diazotrophic bradyrhizobia are often indistinguishable from symbiotic strains [100]. The lack of symbiotic properties may, however, be compensated by other attributes as revealed in bradyrhizobia inhabiting forest soil habitats that are rich in organic matter, whose genomes are enriched in loci involved in the catabolism of aromatic compounds [102].

The colonization of terrestrial environments by land plants had a profound impact on soil microbiota [103]. It is likely that *Bradyrhizobium* benefited from the expansion of land plants, developing a range of, mainly, mutualistic associations, which had been formed prior to the emergence of nitrogen-fixing symbiosis with *Fabaceae*. This explains the persistence of this genus in soils regardless of the presence of legumes [104,105,106]. Remarkably, members of this genus also play important roles in the nitrogen and sulphur cycles [64,107] and are also involved in a range of, mainly, mutualistic interactions with animals [108,109,110].

## 3. *Bradyrhizobium japonicum* and Related Species

**Figure 2:**
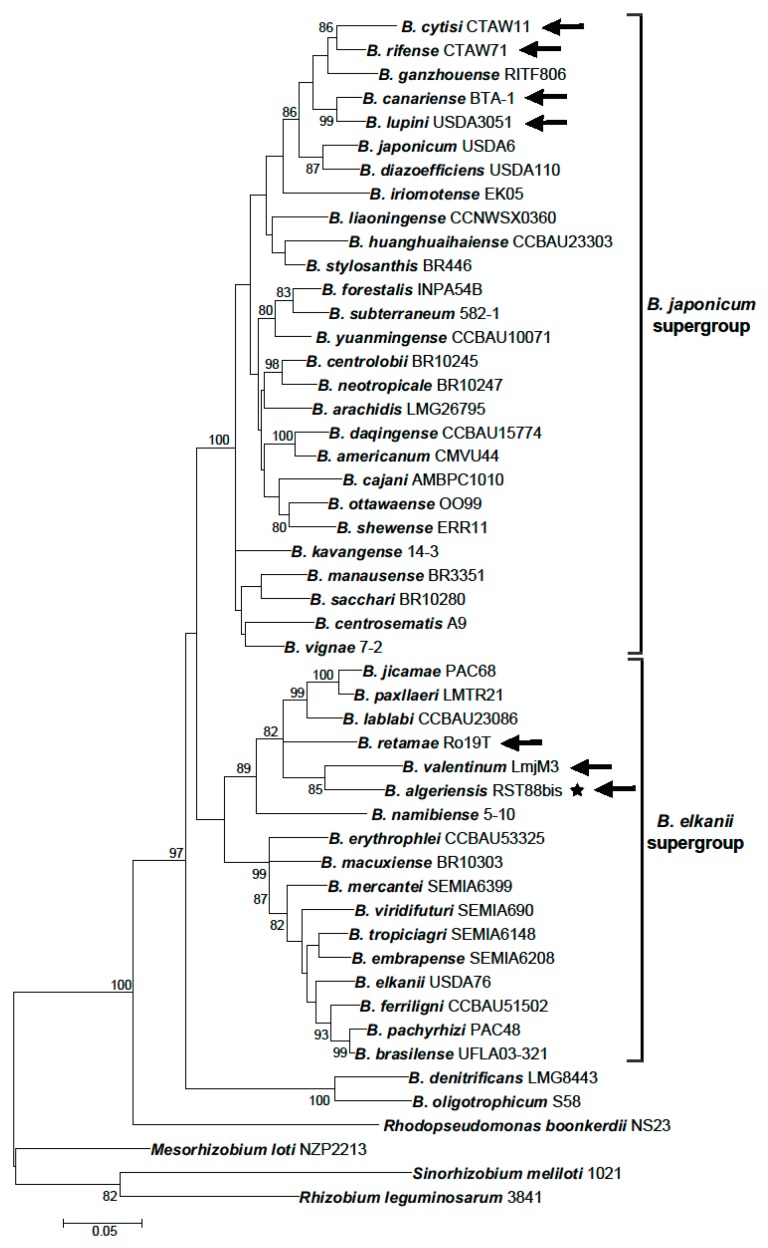
Maximum likelihood (ML) phylogeny of concatenated *recA* and *glnII* partial gene sequences (425 bp + 519 bp), comprising type strains of *Bradyrhizobium* species with the exception of species, in which *recA* sequences were missing: *Bradyrhizobium betae* LMG 21987, *Bradyrhizobium guangdongense* CCBAU 51649, *Bradyrhizobium guangxiense* CCBAU 53363, *Bradyrhizobium icense* LMTR 13 and *Bradyrhizobium ingae* BR 10250. The scale bar indicates the number of substitutions per site. Bootstrap values >70% (percentage of 500 replicates calculated under distance criteria) are given at the branching nodes. The sequences of *Rhodopseudomonas boonkerdii* NS23, *M. loti* NZP2213, *Sinorhizobium meliloti* 1021 and *R. leguminosarum* 3841 were used as outgroups. The sequences were aligned using ClustalW software and ML phylogenies were inferred with Mega 6 [17] using the best-fit nucleotide substitution models as indicated by jModelTest 2.1.4. [115]. The distances were calculated according to the GTR+I+G model. Arrows indicate *Bradyrhizobium* species that nodulate Genisteae plants. Asterisk denotes *Bradyrhizobium algeriensis,* which has not been formally recognized.

Currently, the genus *Bradyrhizobium* comprises 50 species that have been proposed for strains originating from Europe, Northern Africa, South America, and Asia (see Supplementary Table S1). Despite recent efforts that were centred on the characterization of nonsymbiotic bradyrhizobia, type strains of 45 species are bacteria isolated directly from root or stem nodules of *Fabaceae* plants. Importantly, phylogenies of the 16S rRNA gene and several other core (nonsymbiotic) gene markers that are commonly used in MLSA (multiple locus sequence analysis) studies reveal two major branches in the genus *Bradyrhizobium* [111]. One of the branches, referred to as the *B. japonicum* supergroup or *Bradyrhizobium* group I, currently comprises 29 species, in addition to *B. japonicum* [112,113] (Figure 2 and Figures S1 and S2). The remaining *B. elkanii* supergroup or group II contains *B. elkanii* and 17 other species (Supplementary Table S1). *Bradyrhizobium denitrificans* and *Bradyrhizobium oligotrophicum* tend to group separately from the two supergroups. Apart from the 50 recognized species, *Bradyrhizobium* core gene phylogenies uncover many unnamed groups and single-strain lineages [28,114].

Earlier studies suggested that lupin bradyrhizobia are related to, or are often indistinguishable from, many soybean isolates in core gene phylogenetic trees [23,24,35,73,116]. Given that soybean and lupin bradyrhizobia belong to different cross-inoculation groups, two symbiovars (symbiovar genistearum for strains nodulating Genisteae and symbiovar glycinearum for microsymbionts of soybean) were proposed [35,117]. This division enabled the differentiation of related *Bradyrhizobium* strains by referring to their distinct symbiotic properties.

Studies that followed the publication of Barrera et al. [116] suggested that *Bradyrhizobium* isolates of Genisteae spp. showed phylogenetic affinity to *B. japonicum*, i.e., all of the isolates belonged to the *Bradyrhizobium japonicum* supergroup. This observation concerned the isolates originating from Genisteae plants growing in acidic or neutral soils in the Mediterranean and the Andes, the regions encompassing the principal centres of Genisteae differentiation [34,51,60,63,64,79,80]. Also, related *Bradyrhizobium* strains were detected among Genisteae isolates at the northern margin of this tribe’s geographical range in Belgium and Poland [48,58,65]. Likewise, *B. japonicum*-related strains were described in the western part of the United States among *Bradyrhizobium* isolates that were recovered from lupin and *Acmispon* spp. [70,118].

Although the prevalence of *B. japonicum-*related strains among Genisteae isolates was highlighted in earlier work, a closer examination revealed that a number of strains originating from more acid soils (pH <6) clustered separately with respect to *B. japonicum* species. Some strains infecting genistoid legumes in the Canary Islands were subsequently delineated as *Bradyrhizobium canariense* species [35]. It was then shown that *B. canariense* is widespread in the Mediterranean among Genisteae isolates, including several lupin species, *Cytisus aeolicus*, *Genista aspalathoides*, *Retama sphaerocarpa*, and *Spartium junceum* [25,34]. Further studies revealed that this species is also common in the western United States [118,119]. The western part of the United States constitutes a major centre of differentiation of the genus *Lupinus*, moreover, it is also inhabited by multiple Loteae species [12,120,121,122]. Also, *B. canariense* strains were reported in Poland and Iceland as well as in Western Australia in areas where lupin and serradella crops were cultivated [60,65,68]. The identification of *B. canariense* in areas located outside a Mediterranean climate appeared to be associated with acidic soils and suggested that soil pH is a primary factor determining the range of this species.

In the following years, *Cytisus villosus* was found to be infected by *B. canariense* and strains that belonged to two other species, which were broadly related to *B. japonicum* groups. These strains were formally described as *Bradyrhizobium cytisi* and *Bradyrhizobium rifense* [49,52]. This indicated that *C. villosus*, like most Genisteae so far studied, is nodulated by a broad range of *Bradyrhizobium* lineages. *B. cytisi* strains were also identified among both symbiotic and nonsymbiotic isolates which inhabit the rhizosphere of *Acmispon strigosus* (tribe Loteae) sampled across California [118].

Finally, a new species, *B. lupini* was proposed for strain USDA3051 that occupied a sister position with respect to *B. canariense* isolates in 16S rRNA and *glnII*-*recA* phylogenies [69]. This species seems to be common among Genisteae symbionts in the Mediterranean, which could be deduced from its presence together with *B. canariense* among recently recovered *Bradyrhizobium* symbionts of *Lupinus micranthus* and *Lupinus luteus* in Spain, Algeria, and Tunisia [78,79,80]. In addition to the dominant *B. canariense–B. lupini* group, the report of Msaddak et al. [79] unveiled two other, yet unnamed groups within the *B. japonicum* supergroup, one of which (Group II) showed an affinity to *B. betae*, a nonsymbiotic bacterium isolated from sugar beet root [123], while the other (Group V), depending on markers used (*recA* or *glnII-recA-rrs*) clustered with either *B. rifense* or *Bradyrhizobium diazoefficiens* spp.

It appears that *B. canariense*, *B. cytisi*, *B. lupini*, and *B. rifense* spp. prevail among the microsymbionts of Genisteae spp. in acidic and neutral soils. All of these species are probably adapted to poor, oligotrophic soils formed in a semi-arid-mediterranean climate. It is, thus, hardly surprising that these species were detected in regions that are known for a high diversity of Genisteae spp., i.e., in the Mediterranean, and in the western part of the United States. On the other hand, *B. japonicum* seems to be more common in temperate and subtropical climate, associating with legumes that are growing in more neutral soils [24,35,49,51,52,60,65,118,119].

## 4. Genisteae Isolates Related to *Bradyrhizobium elkanii*

Although most *Bradyrhizobium* isolates from Genisteae species belong to the *B. japonicum* supergroup, some cluster in the *B. elkanii* supergroup. Among *Bradyrhizobium* strains isolated from lupins native to Brazil, a strain originating from *Lupinus paraguariensis* aligned within the *B. elkanii* supergroup [60]. Related *Bradyrhizobium* strains were later described among root-nodule isolates that were recovered from *Lupinus albescens* plants [61]. *L. albescens* belongs to the same lupin group as *L. paraguariensis*, showing a similar range encompassing the northern part of Argentina, but also Uruguay, Paraguay, and southern Brazil [4,124]. Like other lupins, *Lupinus albescens* is adapted to poor, sandy soils, although it also inhabits more fertile soils. Out of the three major groups uncovered in core gene phylogenies, one group (Cluster III) [61], comprised strains from non-arenized-non-sandy soils that clustered in proximity to *Bradyrhizobium pachyrhizi-B. elkanii* spp. However, the remaining strains, referred to as Cluster I (contained strains from non-arenized soils) and Cluster II (contained strains from arenized-sandy soils) grouped separately within the *B. japonicum* superclade [61].

A considerable effort was centred on the investigation of rhizobial symbionts of *Lupinus mariae-josephae*. Unlike other lupins, most of which prefer acidic or neutral soils, *L. mariae-josephae* grows in alkaline, calcareous soils. This lupin species is also highly endemic—its geographic range is limited to small populations, scattered across the Valencia province in Eastern Spain [125]. The characterization of *L. mariae-josephae* rhizobium isolates originating from soil samples collected at a single location revealed a substantial level of diversity. All *Bradyrhizobium* strains isolated in this work grew very slowly, and clustered within the *B. elkanii* supergroup [64]. In a subsequent study, a much larger number of *L. mariae-josephae* isolates (103 strains) were characterized, from plants sampled at four principal locations of its natural occurrence. Phylogenies based on combined sequences of *atpD*, *glnII*, and *recA* genes uncovered six major groups (group I-VI), all were confined to the *B. elkanii* supergroup [75]. Three closely related *L. mariae-josephae* isolates (each belonging to group I) were then subjected to detailed molecular and phenotypic analyses, which resulted in the description of a new species, *Bradyrhizobium valentinum* [76]. The systematic status of isolates representing the remaining groups (groups II-VI) is unclear, however, these strains do not group together with known *Bradyrhizobium* species, and thus likely represent separate species within this genus.

Lupin is not the only Genisteae genus infected by strains within the *B. elkanii* supergroup. In fact, strains showing phylogenetic affinity to *B. elkanii* were previously reported in Sicily, among the isolates nodulating *Calicotome spinosa* and *G. aspalathoides* [34]. In addition, *B. elkanii*-related strains were detected in nodules of *Retama raetam* and *R. sphaerocarpa*, in seven distinct ecological-climatic areas of north-eastern Algeria [92]. In another study, all of the *Bradyrhizobium* strains isolated in Morocco and Spain from *R. sphaerocarpa* and *Retama monosperma* clustered in the *B. elkanii* supergroup. Here, isolates were classified as a new species, *Bradyrhizobium retamae*. In the phylogenetic tree based on concatenated sequences of *recA*, *atpD*, and *glnII* genes, *B. retamae* occupied a sister albeit distinct position with respect to *Bradyrhizobium lablabi* and *Bradyrhizobium jicamae* species [88]. Notably, *B. retamae* was the first *Bradyrhizobium* species nodulating Genisteae plants with an affinity to the *B. elkanii* supergroup. Later, for *Bradyrhizobium* isolates that were recovered from *R. sphaerocarpa* plants growing in different soil and climatic conditions in Spain, roughly one-third of the isolates clustered in the *B. japonicum* supergroup, whereas the remaining strains grouped in the *B. elkanii* cluster [93]. Strains belonging to these two major groups of *Bradyrhizobium* were also detected among the symbionts of *Genista versicolor,* which is endemic to the Sierra Nevada National Park in Spain [59]. It is emphasized that a significant proportion of the strains that were isolated in the studies described formed phylogenetic lines clearly distinct from the known *Bradyrhizobium* species, which indicates a very high level of diversity within Genisteae symbionts in the Mediterranean.

## 5. Phylogeny of Symbiotic Loci of Genisteae-Nodulating *Bradyrhizobium* Strains

**Figure 3:**
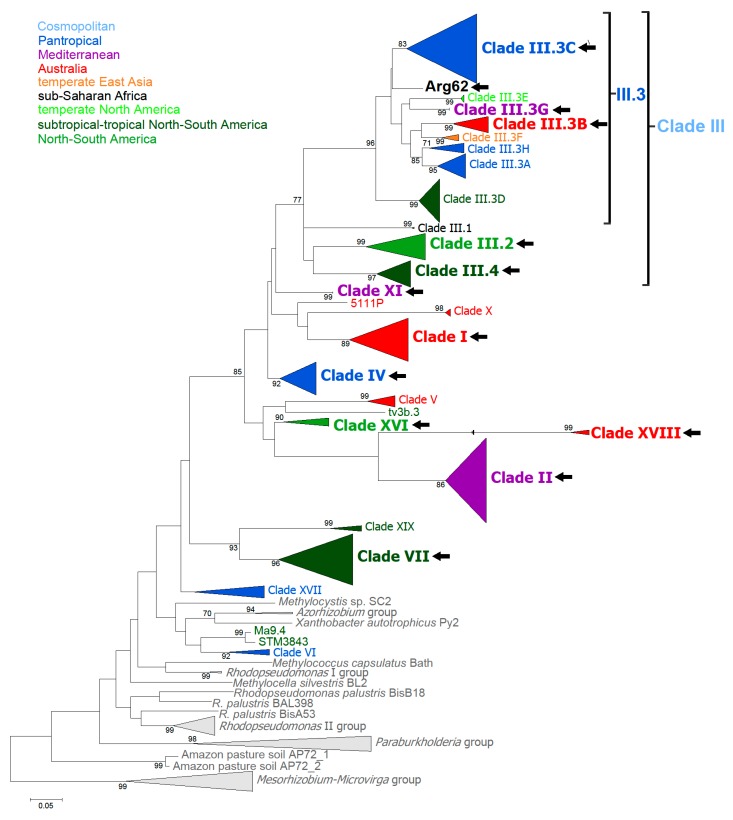
Maximum likelihood (ML) tree based on bradyrhizobial *nifD* gene sequences (759 bp). The significance of each branch is indicated by the bootstrap percentage calculated for 500 bootstraps. The bootstrap values greater than 70% are indicated at nodes. The sequences were aligned using ClustalW software and ML phylogenies were inferred with Mega 6 [17] using the best-fit nucleotide substitution models, as indicated by jModelTest 2.1.4. [115]. The distances were calculated according to the HKY+I+G model. Because of the substitution saturation that is associated with the third codon position in the *nifD* dataset, as estimated using DAMBE 5 [133] these positions were excluded from further analysis. The number of sequences used in the construction of this phylogenetic tree is given in brackets: *Bradyrhizobium*: (640); *Rhodopseudomonas* (29); *Paraburkholderia*/*Burkholderia* (19); *Mesorhizobium* (22); *Microvirga* (3); *Azorhizobium* (2). The number of *Bradyrhizobium* sequences included in a particular clade or branch is also shown in brackets: Clade I (54), Clade II (106), Clade III(III.1) (2), Clade III(III.2) (33), Clade III(III.3A) (31), Clade III(III.3B) (22), Clade III(III.3C) (87), Clade III(III.3D) (54), Clade III(III.3E) (9), Clade III(III.3F) (8), Clade III(III.3G) (3), Clade III(III.3H) (12), Clade III(III.4) (33), Clade IV (41), Clade V (14), Clade VI (7), Clade VII (64), Clade X (9), Clade XI (2), Clade XVI (10), Clade XVIII (6), Clade XIX (7), Clade XX (15). Black arrows indicate *Bradyrhizobium* species nodulating Genisteae plants.

The analysis of *nodA*, *nodC,* as well as *nifD* and *nifH* gene sequences revealed a high level of diversity within the genus *Bradyrhizobium* [21,60,70,82,114,126,127,128,129,130,131]. In recently published phylogenies of *nodA* and *nifD* symbiotic genes, *Bradyrhizobium* strains formed 16 major groups, referred to as Clade I–Clade XVI [28]. Following this classification scheme, *Bradyrhizobium* strains nodulating Genisteae spp. cluster in the *nodA* tree in Clade I, Clade II, Clade III, Clade IV, Clade VII, Clade XI, Clade XIII, Clade XV, and Clade XVI [60,61,65,76,114,132]. In the current work, in phylogenies that were based on sequences of *nodA* and *nifD* genes, Clade V is formed solely by strains originating from Australian native legumes [114]. Some South American strains that previously were assigned to Clade V were transferred to subclade III.4 of Clade III, which in symbiotic gene trees cluster separately with respect to the Australian strains (Figure 3 and Figure S3).

Clade II, also referred to as the lupin clade, was the first described symbiotic *nodA* gene group comprising *Bradyrhizobium* isolates from Genisteae spp. [132]. Further work revealed that Clade II is a dominant group among Genisteae bradyrhizobia in Europe and the Mediterranean [21,39,65,73,78,79,80]. Strains belonging to this group were also detected among *Bradyrhizobium* isolates that were recovered from *Lupinus* spp. endemic to the Andes, but not in lupin species that are native to the eastern part of Brazil [60,61]. Clade II strains also prevail among lupin isolates in North America, especially, in the western part of the United States [70,71,82,134].

The geographical range of Clade II overlaps with the distribution of Genisteae genera in the Mediterranean and the western part of the Americas. The exception is southern Africa where the four basal genera (*Argyrolobium*, *Dichilus*, *Melolobium*, and *Polhillia*) have their centres of divergence [2]. Presumably, these South-African genera are not nodulated by Clade II bradyrhizobia, as suggested by two recent studies [27,28]. However, this opinion should be taken with caution, given that only a limited number of Genisteae isolates from these four genera have so far been characterized in South Africa. Thus, we cannot exclude the possibility that early stages of Clade II differentiation took place in southern Africa, being connected to the divergence of the basal Genisteae genera [135]. In South Africa, Clade II bradyrhizobia have been detected as symbionts of *Lupinus angustifolius* which is exotic to this region. However, these strains were similar to European bradyrhizobia and it was assumed that they had been accidentally brought from Europe together with soil-contaminated lupin seeds [68].

Notably, the geographic range of Clade II to a large extent overlaps with the range of the Loteae tribe (Table S2). This legume tribe has its major centre of divergence in the Mediterranean, extending to western Asia, while the secondary centre of divergence is in the western part of the United States [120,121]. Although the Loteae plants are infected, primarily, by *Mesorhizobium* strains, at least some genera, e.g., the genus *Acmispon* in the western part of the United States, and the genus *Ornithopus* (serradella) in Europe show preference for Clade II bradyrhizobia [65,71,118,134]. Actually, Serradella species that are known to establish highly specific symbiosis with their rhizobial partners have, for a long time, been used as surrogate hosts for isolation of effective strains nodulating lupins and other Genisteae spp. [136,137]. Out of the five *Ornithopus* species, four are native to the Mediterranean and/or western and central Europe, whereas *Ornithopus micranthus* is endemic to south-eastern Brazil, north-eastern Argentina and Uruguay [121]. Evidently, Clade II bradyrhizobia are the preferred symbionts of pink (*Ornithopus sativus*) and yellow serradella (*Ornithopus compressus*) species [35,64,65,68]. Much less is known about rhizobia nodulating *Ornithopus perpusillus* and *Ornithopus pinnatus*, and the south American *O. micranthus* sp., although the two European species seem to be also nodulated by Clade II *Bradyrhizobium* symbionts [48,65,138]. Like the four European species, *O. micranthus* is infected by *Bradyrhizobium* strains, however, nothing is known about the phylogenetic affinities of symbiotic loci in these strains [139].

It has been assumed that Clade II *Bradyrhizobium* has evolved in the Mediterranean and that this process has been somehow connected to the diversification of Genisteae [60]. In fact, when considering the number of Genisteae genera, the Mediterranean is the major (although not primary) centre of divergence of this tribe [10,140,141]. The idea of coevolution of bradyrhizobia with Genisteae diversifying in the Mediterranean seems plausible as bradyrhizobia originating from this region occupy the outermost position within the *nifD* tree shown in Figure S4. However, the majority of Clade II *nifD* sequences form two internal branches, one of which comprises the “European” while the other the “American” isolates (Figure S4). It has to be mentioned that the “European” branch also includes bradyrhizobia that were recovered in the United States [44] and all American strains in this branch originate from *Cytisus scoparius* and are indistinguishable from European Clade II isolates. This is in line with a recent study [134], which revealed that European Genisteae legumes introduced to the United States show preference for “European” Clade II bradyrhizobia even in areas inhabited by native legumes nodulated by “American” Clade II strains.

Importantly, this branching pattern corroborates the assumption that the Mediterranean is the initial centre of Clade II divergence whereas the western part of the United States is a secondary centre of divergence (Figure 3 and Figure S4). The higher specificity of symbiosis with the Loteae and the fact that geographic ranges of these two tribes largely overlap indicate that the Loteae may have been a major driver in Clade II evolution. According to the “Jack-of-all-trades is master of none” hypothesis, one can assume that a specialist (in this case Clade II strains) shows much better ‘fitness’ with respect to a “generalist” group nodulating Genisteae [71]. Thus, the ability to form highly effective symbiosis with the two legume tribes (one of which is more restrictive) could explain the dissemination of Clade II bradyrhizobia across the Mediterranean and temperate parts of the Americas.

Clade III is the most heterogeneous and cosmopolitan group and it also shows the broadest host range amongst *Bradyrhizobium* major clades (see Table S2). In the *nifD* phylogenetic tree, this branch forms four, well supported inner branches referred to as III.1, III.2, III.3, and III.4 (see Figure 3 and Figure S3). Subclade III.1 comprises only two isolates from Senegal, while the remaining three subgroups are more numerous, containing bradyrhizobia originating from Genisteae (from American lupins) (Table S2). Unlike the cosmopolitan subclade III.3, *Bradyrhizobium* strains belonging to subclades III.2 and III.4 were detected solely in the Americas. Nevertheless, several independent reports indicate that these two groups are probably common among rhizobia infecting lupins that are native to South and North America [21,60,61,70,82]. In III.2 and III.4 subclades, bradyrhizobia originate from legumes belonging to 10 and 9 tribes, respectively, which implies that, although they have diversified in the Americas, there is a lack of evidence supporting a lasting co-evolution with Genisteae spp. (Table S2).

The largest and most cosmopolitan subclade III.3 comprises eight internal branches (III.3A-III.3H) (Figure 3). *Bradyrhizobium* strains originating from Genisteae are confined to branches III.3B, III.3C, and III.3G. The small branch III.3G contains identical *nifD* sequences from *C. scoparius* isolates originating from Spain and the United States, which suggests that this group may have a Mediterranean origin. In the predominantly Australian branch III.3B, four isolates originating from Spain (two strains from *R. sphaerocarpa*) and Portugal (two strains, one from introduced *Acacia longifolia* and one from native *Cytisus grandiflorus*) form an outer group. It cannot, however, be excluded that these strains, albeit originally from Australia, have extended their host range to the native Mediterranean genera [95]. On the other hand, the diverse branch III.C contains a single isolate from *Argyrolobium rupestre* (strain Arg105), originating from South Africa [28]. The branch III.C comprises the isolates from sub-Saharan Africa, southern Asia, Australia, as well as from Central and North Americas, therefore it can rightly be regarded as a cosmopolitan group.

*Bradyrhizobium* Clade IV strains nodulating Genisteae have been reported in the Mediterranean (in Algeria, Croatia, Italy, and Spain), among rhizobial symbionts of *C. spinosa*, *G. aspalathoides*, *Laburnum anagyroides*, *L. mariae-josephae*, *R. monosperma*, *R. sphaerocarpa,* and *S. junceum* [21,34,75,76]. Some of the isolates from *L. mariae-josephae* and *Retama* spp. belong to *B. valentinum* and *B. retamae*. Moreover, Clade IV bradyrhizobia were assigned to *Bradyrhizobium icense*, *B. lablabi*, *Bradyrhizobium namibiense*, *Bradyrhizobium paxllaeri*, and some as yet unnamed lineages (see Supplementary data Tables S1 and S2). This group shows a broad geographic range encompassing mainly arid and semi-arid parts of Africa, Asia, Australia, Europe, and the Americas [68,114,129,130,142].

Highly differentiated Clade VII comprises *B. americanum*, *Bradyrhizobium ingae*, *B. iriomotense*, *B. manausense*, and *B. stylosanthis* spp. (Supplementary data Table S1). The majority of Clade VII strains originate from North and South America, while some were isolated in south-east Asia (southern China, the Island of Okinawa, Thailand, and the Philippines) [143,144,145,146,147]. Given that Clade VII bradyrhizobia from south-east Asia occupy internal positions relative to the American strains, it can be assumed that this cluster has evolved in the Americas, possibly, in an area of hot and humid tropical climate. This can be deduced from the fact that this clade is common in tropical-humid (but not arid) parts of Central and South America, and the Caribbean [21,53,60,70,82]. Clade VII strains nodulate a broad range of, mainly, tropical legumes (Table S1), however, some strains originate from lupins that are endemic to south-eastern Brazil [60,61]. Like Clade VII, Clade XVI seems to be an American group. So far, *Bradyrhizobium* strains belonging to Clade XVI have been described among legume isolates from Costa Rica, Honduras, the Caribbean (the Island of Guadeloupe), and the United States [53,148,149,150]. Unlike Clade XVI strains that were isolated in Central America and the Caribbean which do not originate from Genisteae spp., all Clade XVI strains from the United States originate from *Lupinus lepidus*, *Lupinus perennis*, and *A. strigosus* [70].

In the *nodA* tree (Figure S3), *Bradyrhizobium* strains Arg62, Arg68, and Arg33 from the South African *Argyrolobium sericeum* cluster in Clade XV. This clade also comprises the isolates from *Lotononis* and *Pearsonia* spp.—the Crotalarieae genera endemic to southern Africa [1,28,151]. The grouping of these strains in the same cluster is not surprising considering the overlapping geographical ranges of the southern Genisteae genera and the Crotalarieae tribe in South Africa [27]. However, in the *nifD* tree (Figure 3), strain Arg62 groups within Clade III.3, while Arg33 has been included in Clade IV. This indicates that although *nodA* and *nifD* phylogenies are essentially congruent, there are some differences, most likely reflecting distinct evolutionary histories of these two symbiotic genes in particular clades.

Clade I has been described as a predominant group in temperate and tropical Australia, where this group of bradyrhizobia nodulate native legumes, primarily, members of the endemic Bossiaeeae-Mirbelieae tribes and the genus *Acacia* [68,114,132]. In addition, Clade I strains have been isolated outside this continent, usually from the introduced Australian *Acacia* species [95,152,153]. This implies that Clade I bradyrhizobia may have been co-introduced with *Acacia* spp. and that in their new habitats they out-competed the indigenous rhizobia in the process of nodulation of their native hosts. Importantly, Clade I bradyrhizobia were also reported in nodules of *C. grandiflorus*, *L. micranthus*, and *Ulex europaeus* (gorse), which can be regarded as an extension of Clade I strains’ range on native-Mediterranean hosts. This phenomenon of rapid adaptation to new legume hosts may be widespread in the genus *Bradyrhizobium* [80,95,154]. This can be concluded from the identification of other, presumably Australian groups, e.g. group III.3B (which has been mentioned above) as well as an enigmatic Clade XVIII (Figure 3). Clade XVIII is a phylogenetically distinct group, comprising *nifD* sequences (there is a lack of *nod* gene sequences for Clade XVIII in the GenBank database) which originate from *Bradyrhizobium* strains isolated from *A. longifolia* and *A. saligna* in Australia and *A. saligna* and *Cytisus* sp. in Portugal [155]. Although the Mediterranean origin of this group cannot be excluded, the association with Australian *Acacia* spp. in both Australia and in areas in Portugal that are infested by these mimosoid spp. strongly suggests that Clade XVIII has an Australian origin.

## 6. Fast-Growing Rhizobium Genera

In comparison to the genus *Bradyrhizobium*, much less is known about members of the fast-growing rhizobial genera that infect Genisteae plants. Nonetheless, work over the last 10 years has shown that Genisteae legumes are nodulated by both highly cosmopolitan genera as well as genera that occur rarely among rhizobial isolates.

The genera *Mesorhizobium*, *Rhizobium,* and *Ensifer* (=*Sinorhizobium*), along with *Bradyrhizobium* are regarded as the most cosmopolitan rhizobium groups due to their ability to nodulate a broad range of *Fabaceae* spp. [156]. However, available data indicate that these genera are not common symbionts of Genisteae species [156]. *Mesorhizobium* strains classified as *M. loti* nodulate with lupins, although they show a preference for *Lotus* spp., which, presumably, are their primary hosts. It seems, however, that *M. loti* is not the only *Mesorhizobium* species that is involved in root-nodule symbiosis with Genisteae. Recently, two *Mesorhizobium* strains isolated from North American lupins, one from *Lupinus densiflorus,* and the other from *L. succulentus*, were shown to have a phylogenetic affinity to *Mesorhizobium ciceri*—a species that until now has not been implicated in the symbiosis with genistoid legumes [157]. In the Mediterranean, *Mesorhizobium* strains nodulate with native Genisteae spp, including *Genista saharae*, *R. raetam*, *Teline monspessulana*, and three lupin species (see Table 1). However, only limited information is available about these strains especially their symbiotic loci [34,55,56,62]. *Mesorhizobium* strains were also recovered from root nodules of *Argyrolobium lunare* and *A*. *velutinum* in the Core Cape Subregion (the Fynbos) of South Africa. Interestingly, these isolates formed discrete branches on core gene and *nodA* phylogenetic trees, grouping together with strains nodulating *Asphalathus* spp. (tribe Crotalarieae), which indicates that these two genera are nodulated by closely related bacteria that may potentially form a single cross-inoculation group [27].

Although the isolation of *Rhizobium* strains from lupin nodules has been reported in a number of studies, to our knowledge the symbiotic effectiveness of these isolates on lupins has not been confirmed. Usually, strains described as *Rhizobium*, have not been sufficiently characterised at the molecular level or authentication tests on their original host have not been performed or proved negative [157,158,159]. For example, strain H 13-3 originally described as *Rhizobium lupini*, following more detailed analysis was reclassified as *Agrobacterium* sp. [160]. Also, two strains recently isolated from lupin species native to Morocco have been assigned to the genus *Rhizobium* based on the ARDRA analysis [161]. These strains require further characterization of selected core and symbiotic marker genes [111]. Nonetheless, there are reports indicating that members of the genus *Rhizobium* nodulate other Genisteae genera, including *Adenocarpus* spp., *Argyrolobium uniflorum*, *C. spinosa*, *Cytisus* spp. *Genista* spp., and *R. raetam* (see Table 1). However, as in the case of the lupin isolates, more detailed molecular characterization and authentication tests are needed to confirm their phylogenetic affinity and their symbiotic properties.

*Ensifer* (*Sinorhizobium*) strains have not been described in lupin nodules, which contrasts with several other Genisteae spp. that are nodulated by rhizobia belonging to this genus (see Table 1). For instance, *Argyrolobium uniflorum* and *Genista saharae* are infected exclusively by fast-growing rhizobium genera, in particular, *Ensifer* [29,30,31,55,56,91]. In the case of these two species, the predominance of *Ensifer* as symbiont may be caused by specific requirements of these particular Genisteae species, favouring this rhizobium genus. However, it may be related to the high salt and arid habitat of the legumes, which may favour *Ensifer* in comparison to other rhizobial genera [162].

In addition, to the four genera described above, Genisteae spp. are infected by three other fast-growing rhizobial genera. In 2005, Trujillo and co-workers described rhizobia classified as *Ochrobactrum lupini*, which were isolated in Argentina from native *Lupinus honoratus* [72]. Prior to this finding, this genus was assumed to comprise opportunistic pathogens and saprophytes living in soil and animal faeces [163]. Subsequently, *Ochrobactrum* strains were isolated in Spain from nodules of *C. scoparius* (Scotch broom) plants [47]. Also, *Ochrobactrum* rhizobia were described in root nodules of *Acacia mangium* in the Philippines and Thailand [164], as well as in nodules of *Cicer arietinum* in Pakistan [165].

The genus *Microvirga* which comprises soil and water saprophytes was included in the alpha-proteobacterial lineage of root-nodule bacteria only in 2012, although the first symbiotic strains were detected in nodules of *Lupinus texensis* in 2007 [87,166,167]. Recently, *Microvirga* strains were isolated from *L. micranthus* and *L. luteus* spp. in Tunisia, as well as from *L. subcarnosus* in the United States [79,80,157]. This indicates that *Microvirga* may be rather common among lupin isolates in both the Mediterranean and North America. It is unclear if *Microvirga* can nodulate Genisteae spp. other than lupins, however, aside from lupins, rhizobia belonging to this genus were also isolated from *Listia angolensis* in Zambia, *Vigna* sp. in Brazil, and from *Vicia alpestris* in the Caucasus, Russia [87,168,169].

The *Phyllobacterium* genus comprises bacteria that are well-known for their epiphytic and endophytic associations with plants [170]. Some phyllobacteria fix nitrogen, therefore, the occurrence of root-nodule bacteria in this genus is not unexpected [161]. Indeed, an isolate classified as *Phyllobacterium trifolii* was found nodulating white lupin (*Lupinus albus*) in addition to its original white clover (*Trifolium repens*) host. However, *P. trifolii* strains formed ineffective nodules on white lupins, which indicated that lupins are not their natural hosts [171]. Nonetheless, *Phyllobacterium* rhizobia were described in Tunisia, in the nodules of *L. micranthus* [79]. Prior to this finding, *Phyllobacterium* strains were identified as symbionts of *Adenocarpus hispanicus*, *Genista saharae* and *G. tinctoria*, *R. sphaerocarpa*, and *S. junceum* (see Table 1). This genus may be specialized in the nodulation of genistoid legumes, however, it cannot be excluded that some of the *Phyllobacterium* isolates are endophytic bacteria that are lacking the ability to form nodules [172].

The data discussed indicate that although members of the genera *Microvirga*, *Ochrobactrum*, and *Phyllobacterium* nodulate various legume-hosts (including some members of the tribe Genisteae), and show broad geographical range, they most likely prevail among root-nodule bacteria in only discrete environments [156]. While all the Genisteae isolates characterised so far are α–rhizobia, this may change if authentication tests confirm symbiotic-nitrogen-fixing properties of recently described lupin isolates belonging to the genus *Burkholderia* [157].

## 7. Summary

The high level of diversity shown by Genisteae microsymbionts most likely reflects the complex evolutionary history of this legume tribe, which can be linked to long-distance dispersal and radiation events in southern Africa, the Mediterranean, and the Americas. One can assume that following the dispersal, rhizobial communities which were encountered in newly colonized areas often differed from rhizobial symbionts in the areas of Genisteae primary occurrence. The lack of appropriate rhizobial symbionts is often perceived as an obstacle that is impeding the dispersal of particular *Fabaceae* spp. [173]. However, *Fabaceae* spp. may interact with indigenous rhizobia forming less efficient symbiosis [174,175]. This “opportunistic” strategy of the two symbiotic partners results in a broader range of rhizobial symbionts, and may explain why certain rhizobial genera cannot be regarded as optimal partners of their legume hosts. In the case of Genisteae spp., the necessity of adaptation to local rhizobia is manifested by the formation of symbiotic associations with members of at least seven rhizobial genera, out of the 16 genera that are known for their symbiotic nitrogen-fixation ability.

Despite the progress that has been made in the last ten years in research focused on the fast-growing genera nodulating Genisteae spp., the current knowledge concerning this diverse group of rhizobia lags significantly behind the understanding of the symbiosis that is established between Genisteae spp. and their *Bradyrhizobium* symbionts. Nonetheless, these efforts revealed three new genera, out which *Microvirga* appears to comprise effective symbionts of at least some lupin species in North America and the Old World [79,80,167].

Unlike the fast-growing rhizobia, the genus *Bradyrhizobium* appears to be a dominant group nodulating the majority of Genisteae species. Genisteae-nodulating bradyrhizobia cluster within both *B. japonicum* and *B. elkanii* superclades, belonging to seven distinct species, and a large number of partially characterized lineages. In symbiotic gene phylogenies, *Bradyrhizobium* symbionts are scattered in several distinct groups, each comprising strains originating from phylogenetically distinct legumes. This indicates that the capacity for nodulation of Genisteae spp. appeared independently in various symbiotic gene clades, and that the adaptation towards nodulation of this tribe was not a multi-step process. We assume that this process could be related to the loss of the *noeI* gene, which is involved in the methylation of the fucose moiety at the Nod factor reducing end [176], and which seems to be in a form of pseudogene in *Bradyrhizobium* strains nodulating Genisteae whose genomic sequences are available [60,73,86,177]. The exception could be *Bradyrhizobium* Clade II, which unlike other clusters comprises strains that appear to be specialized in the nodulation of Genisteae, but also Loteae species. It can be presumed that Clade II is an example of the long co-evolution process of Genisteae and their bradyrhizobial symbionts, although the tribe Loteae also may have played an important role.

## Figures and Tables

**Table 1:** Genisteae species and their rhizobium symbionts.

Tribes and Genera	Geographical Origin	Rhizobium Symbionts and Sources
Genisteae		
*Adenocarpus*		
*Adenocarpus decorticans*	Morocco	*Rhizobium* [22]
*Adenocarpus foliolosus*	Spain-Canary Islands	*Bradyrhizobium* [23,24]
*Adenocarpus hispanicus*	Spain	*Phyllobacterium* [25]; *Bradyrhizobium* [26]
*Argyrolobium*		
*Argyrolobium lunare*	South Africa	*Mesorhizobium* [27]
*Argyrolobium rupestre*	South Africa	*Bradyrhizobium* [28]
*Argyrolobium sericeum*	South Africa	*Bradyrhizobium* [28]
*Argyrolobium velutinum*	South Africa	*Mesorhizobium* [27]
*Argyrolobium uniflorum*	Senegal, Tunisia	*Rhizobium*, *Sinorhizobium* (=*Ensifer*) [29,30]; *Sinorhizobium* [31,32]; *Rhizobium* [33]
*Calicotome*		
*Calicotome infesta*	Italy	*Bradyrhizobium* [34]
*Calicotome spinosa*	Italy	*Bradyrhizobium*, *Rhizobium* [34]
*Chamaecytisus*		
*Chamaecytisus proliferus*	Morocco, Spain-Canary Islands, New Zealand	*Bradyrhizobium* [23,24,35,36,37,38]
*Chamaecytisus ratisbonensis*	Poland	*Bradyrhizobium* [39]
*Chamaecytisus ruthenicus*	Russia	*Bradyrhizobium* [40]
*Cytisus*		
*Cytisus aeolicus*	Italy	*Bradyrhizobium* [34]
*Cytisus arboreus*	Morocco	*Rhizobium* [22]
*Cytisus balansae*	Spain	*Bradyrhizobium* [41,42,43]
*Cytisus grandiflorus*	Portugal	*Bradyrhizobium* [21,44]
*Cytisus laburnum*	Spain	*Bradyrhizobium* [25]
*Cytisus multiflorus*	Spain	*Bradyrhizobium* [41,42,43]
*Cytisus proliferus*	Spain-Canary Islands	*Bradyrhizobium* [21,44]
*Cytisus purgans*	Spain	*Agrobacterium, Rhizobium* [25]
*Cytisus scoparius*	Belgium, Ireland, Poland, Spain, UK, USA; Australia, New Zealand	*Bradyrhizobium* [21,37,38,39,41,44,45]; *Bradyrhizobium*, *Mesorhizobium*, *Rhizobium* [46]; *Ochrobactrum cytisi* [47]; *Bradyrhizobium*, *Ensifer*, *Rhizobium*, *Phyllobacterium* [48]
*Cytisus striatus*	Spain	*Bradyrhizobium* [39,42]
*Cytisus triflorus*	Algeria, Morocco	*Bradyrhizobium* [49,50]
*Cytisus villosus*	Algeria, Morocco	*Bradyrhizobium* [49,50,51,52,53]
*Genista*		
*Genista aspalathoides*	Italy	*Bradyrhizobium* Clade II and Clade IV [34]
*Genista germanica*	Poland	*Bradyrhizobium* [39]
*Genista hystrix*	Spain	*Bradyrhizobium* [41,42]
*Genista linifolia*	Spain	*Bradyrhizobium* [54]
*Genista monspessulana*	Spain	*Bradyrhizobium* [54]
*Genista saharae*	Algeria, Tunisia	*Ensifer*, *Phyllobacterium*, *Mesorhizobium*, *Rhizobium* [55]; *Ensifer* [31]; *Ensifer*, *Mesorhizobium*, *Neorhizobium* [56]
*Genista stenopetala*	Spain-Canary Islands	*Bradyrhizobium* [21,44]
*Genista sylvestris*	Croatia	*Bradyrhizobium* [21]
*Genista tinctoria*	Poland, Russia, Slovenia	*Bradyrhizobium* [21,57,58]; *Phyllobacterium, Rhizobium, Bradyrhizobium* [40]
*Genista versicolor*	Spain	*Bradyrhizobium* [59]
*Laburnum*		
*Laburnum anagyroides*	Belgium, Croatia	*Bradyrhizobium* [21,25]; *Bradyrhizobium* [48]
*Lupinus*		
*Lupinus albescens*	Brazil	*Bradyrhizobium* [60,61]
*Lupinus albus*	Poland, Spain	*Mesorhizobium* [62,63]; *Bradyrhizobium* [44,60,64,65,66,67]
*Lupinus angustifolius*	Australia, Poland, South Africa, Spain	*Bradyrhizobium* [60,64,65,68,69]
*Lupinus arboreus*	USA-California, New Zealand	*Bradyrhizobium* [37,70,71]
*Lupinus bandelierae*	Bolivia	*Bradyrhizobium* [60]
*Lupinus bicolor*	USA-California	*Bradyrhizobium* [71]
*Lupinus bracteolaris*	Brazil	*Bradyrhizobium* [60]
*Lupinus breviscapus*	Bolivia	*Bradyrhizobium* [60]
*Lupinus campestris*	Mexico	*Bradyrhizobium* [60]
*Lupinus cosentinii*	Australia, Spain	*Bradyrhizobium* [64,68]
*Lupinus hispanicus*	Spain	*Bradyrhizobium* [64]
*Lupinus honoratus*	Argentina	*Ochrobactrum* [72]
*Lupinus lepidus*	USA-Washington	*Bradyrhizobium* [53,70]
*Lupinus leucophyllus*	USA-Washington	*Bradyrhizobium* [70]
*Lupinus luteus*	Poland, Spain, USA	*Bradyrhizobium*, *Mesorhizobium* [73]; *Bradyrhizobium* [64,65,69]
*Lupinus mariae-josephae*	Spain	*Bradyrhizobium* [64,74,75,76,77]
*Lupinus micranthus*	Algeria, Spain, Tunisia	*Bradyrhizobium* [64,78]; *Bradyrhizobium, Microvirga, Phyllobacterium* [79,80]
*Lupinus misticola*	Peru	*Bradyrhizobium* [60]
*Lupinus montanus*	Mexico-Morelos	*Bradyrhizobium* [24]
*Lupinus mutabilis*	Ecuador	*Bradyrhizobium* [60]
*Lupinus nootkatensis*	USA-Alaska	*Bradyrhizobium* [60,70]
*Lupinus paraguariensis*	Brazil	*Bradyrhizobium* [60]
*Lupinus paranensis*	Brazil	*Bradyrhizobium* [60]
*Lupinus perennis*	USA	*Bradyrhizobium* [81,82]
*Lupinus polyphyllus*	Belgium, Germany, New Zealand, Poland	*Bosea* [83]; *Bradyrhizobium* [24,60,84,85]; *Bradyrhizobium*, *Rhizobium* [48]
*Lupinus pycnostachys*	Bolivia	*Bradyrhizobium* [60]
*Lupinus rubriflorus*	Brazil,	*Bradyrhizobium* [60]
*Lupinus sericeus*	USA-Washington	*Bradyrhizobium* [70]
*Lupinus simulans*	Mexico-Oaxaca	*Bradyrhizobium* [53,70]; *Bradyrhizobium* [53]
*Lupinus succulentus*	USA-California	*Mesorhizobium* [86]
*Lupinus texensis*	USA-Texas	*Microvirga* [87]
*Lupinus tominensis*	Bolivia	*Bradyrhizobium* [60]
*Lupinus uleanus*	Brazil	*Bradyrhizobium* [60]
*Retama*		
*Retama monosperma*	Algeria, Morocco, Spain	*Bradyrhizobium* [88,89]
*Retama raetam*	Algeria, Tunisia	*Agrobacterium*, *Mesorhizobium*, *Rhizobium*, *Sinorhizobium* (*Ensifer*) [90,91]; *Bradyrhizobium* [92]; *Sinorhizobium* [31]
*Retama sphaerocarpa*	Algeria, Morocco, Spain	*Bradyrhizobium* [21,41,88,92,93]; *Bradyrhizobium*, *Phyllobacterium* [25]
*Spartium*		
*Spartium junceum*	Croatia, Italy, Slovenia, Spain	*Bradyrhizobium* [21,34,50,94]; *Bradyrhizobium*, *Phyllobacterium* [25]
*Teline*		
*Teline canariense*	Spain	*Bradyrhizobium* [23,24,35]
*Teline monspessulana*	Italy	*Mesorhizobium* [34]
*Teline stenopetala*	Spain	*Bradyrhizobium* [34]
*Ulex*		
*Ulex europaeus*	New Zealand, Portugal	*Bradyrhizobium* [38,44,48,95,96]

## Supplementary Materials

The following are available online at http://www.mdpi.com/2073-4425/9/3/163/s1. Figure S1: ML phylogeny of *Bradzrhyiobium glnII* gene; Figure S2: ML phylogeny of *Bradzrhyiobium recA* gene; Figure S3: ML phylogeny of *Bradzrhyiobium nodA* gene; Figure S4: The portion of *nifD* ML phylogenetic tree referring to *Bradyrhizobium* Clade II branch; Table S1: The list of *Bradyrhizobium* species; Table S2: The list of Legume genera belonging to *nifD* gene Clades that comprise Genisteae *Bradyrhizobium* symbionts.
