# Supplementary material for: Phylogeny and Phylogeography of Rhizobial Symbionts Nodulating Legumes of the Tribe Genisteae
Source: Genes (Basel). 2018 Mar 14;9(3):163. doi: 10.3390/genes9030163 (PMC5867884; doi:10.3390/genes9030163)
Supplement: Supplementary file 1 [file genes-09-00163-s001.docx]

**Supplementary Materials**

**Figure 1S.** Maximum likelihood (ML) phylogeny of *glnII* partial gene sequences (519 bp), comprising the type strains of all *Bradyrhizobium* species that are known to science. The scale bar indicates the number of substitutions per site. Bootstrap values >70% (percentage of 500 replicates calculated under distance criteria) are given at the branching nodes. Partial *glnII* sequences of *Rhodopseudomonas boonkerdii* NS23, *Mesorhizobium loti* NZP2213, *Sinorhizobium meliloti* 1021 and *Rhizobium leguminosarum* 3841 were used as outgroups. The sequences were aligned using ClustalW software and ML phylogenies were inferred with Mega 6 [17] using the best-fit nucleotide substitution models as indicated by jModelTest 2.1.4. [115]. The distances were calculated according to the GTR+I+G model. Arrows indicate *Bradyrhizobium* species that nodulate Genisteae plants. Asterisk denotes *B. algeriensis* which has not been formally recognized.

**Figure 2S.** Maximum likelihood (ML) phylogeny of *recA* partial gene sequences (425 bp), comprising type strains of *Bradyrhizobium* species with the exception of five species, in which *recA* sequences of appropriate length were missing: *B. betae* LMG 21987, *B. guangdongense* CCBAU 51649, *B. guangxiense* CCBAU 53363, *B. icense* LMTR 13, *B. ingae* BR 10250. The scale bar indicates the number of substitutions per site. Bootstrap values >70% (percentage of 500 replicates calculated under distance criteria) are given at the branching nodes. Partial *recA* sequences of *Rhodopseudomonas boonkerdii* NS23, *Mesorhizobium loti* NZP2213, *Sinorhizobium meliloti* 1021 and *Rhizobium leguminosarum* 3841 were used as outgroups. The sequences were aligned using ClustalW software and ML phylogenies were inferred with Mega 6 [17] using the best-fit nucleotide substitution models as indicated by jModelTest 2.1.4. [115]. The distances were calculated according to the GTR+I+G model. Arrows indicate *Bradyrhizobium* species that nodulate Genisteae plants. Asterisk denotes *B. algeriensis* which has not been formally recognized.

**Figure 3S.** Maximum likelihood (ML) phylogeny of *Bradyrhizobium* *nodA* gene sequences (609 bp). The scale bar indicates the number of substitutions per site. Bootstrap values >70% (percentage of 500 replicates calculated under distance criteria) are given at the branching nodes. The sequences were aligned using ClustalW software and ML phylogenies were inferred with Mega 6 [17] using the best-fit nucleotide substitution models as indicated by jModelTest 2.1.4. [115]. The distances were calculated according to the HKY+I+G model. Because of the substitution saturation was associated with third codon position in the *nodA* dataset, as estimated using DAMBE 5 [133], these positions were excluded from further analysis. Black arrows indicate *Bradyrhizobium* species nodulating Genisteae plants.

**
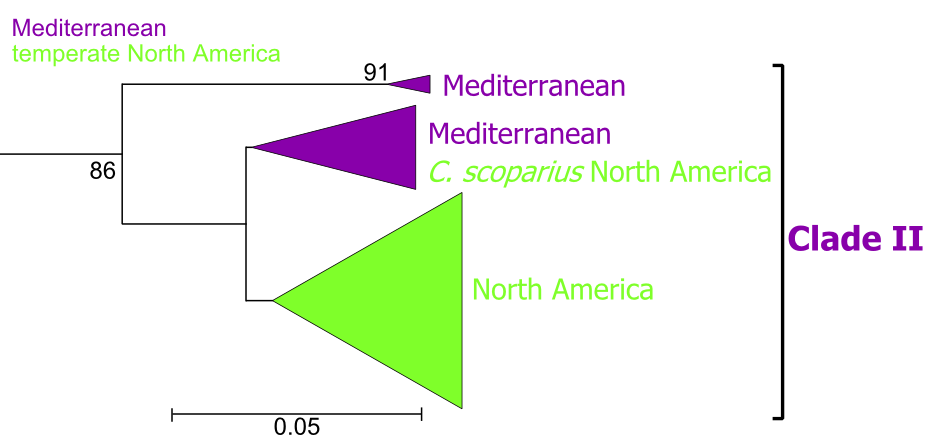
**

**Figure 4S.** The portion of *nifD* maximum likelihood (ML) phylogenetic tree referring to Clade II branch. The scale bar indicates the number of substitutions per site. Bootstrap values >70% (percentage of 500 replicates calculated under distance criteria) are given at the branching nodes.

**Table S1.** The list of *Bradyrhizobium* species.

| **Species name** | **Type strain** | **Genomes** | **Legume host** | **Origin (country)** | **Geographic distribution deduced upon the symbiotic genes** | **Supergroup** | **Symbiotic group (clade)** | **References** |
| --- | --- | --- | --- | --- | --- | --- | --- | --- |
| *B. americanum* | CMVU44^T^ (LMG 29514^T^ = CECT 9096^T^ | - | *Centrosema macrocarpum* | Venezuela | N.-S. America South-east Asia | *B. japonicum* | Clade VII | 147 |
|  |  |  |  |  |  |  |  |  |
| *B. arachidis* | CCBAU 051107^T^ LMG26795T | 1 | *Arachis hypogaea* | China | Pantropical | *B. japonicum* | Clade III (III.3) | 179 |
| *B. betae* | LMG 21987^T^ | - | *Beta vulgaris –tumor-like outgrowth* | Spain | - | *B. japonicum* | Lacks nodulation genes | 123 |
| *B. brasilense* | UFLA03-321^T^ | 1 | *Vigna unguiculata* | Brazil | Pantropical | *B. elkanii* | Clade III (III.3) | 180 |
| *B. cajani* | AMBPC1010^T^ | - | *Cajanus cajan* | Dominican Republic | Pantropical | *B. japonicum* | Clade III (III.3) | 181 |
| *B. canariense* | BTA-1 | 2 | *Chamaecytisus proliferus* | Spain – Canary Islands | Mediterranean, Europe, temperate N.-S. America | *B. japonicum* | Clade II | 35 |
| *B. centrolobii* | BR 10245^T^ | 1 | *Centrolobium paraense* | Brazil | Tropical S. America | *B. elkanii* | Clade XIX | 182 |
| *B. centrosemae* | A9^T^ (LMG 29515^T^ = CECT 9095^T^) | 1 | *Centrosema molle* | Venezuela | Pantropical | *B. japonicum* | Clade III (III.3) | 147 |
| *B. cytisi* | CTAW11^T^ | - | *Cytisus villosus* | Morocco: Rif Mountains | Mediterranean, Europe, temperate N.-S. America | *B. japonicum* | Clade II | 49 |
| *B. daqingense* | CCBAU 15774^T^ | 1 | *Glycine max* | China | Pantropical | *B. japonicum* | Clade III (III.3) | 183 |
| *B. denitrificans* | LMG 8443^T^ | - | *-* | Germany | - | *B. japonicum* | Non-nodulating | 184 |
| *B. diazoefficiens* | USDA 110^T^ | 2 | *Glycine max* | Japan | Pantropical | *B. japonicum* | Clade III (III.3) | 185 |
| *B. elkanii* | USDA 76^T^ | 1 | *Glycine max* | USA | Pantropical | *B. elkanii* | Clade III (III.3) | 186 |
| *B. embrapense* | SEMIA 6208^T^ | 1 | *Desmodium heterocarpon* | Brazil | Pantropical | *B. elkanii* | Clade III (III.3) | 187 |
| *B. erythrophlei* | CCBAU 53325^T^ | - | *Erythrophleum fordii* | China | Pantropical | *B. elkanii* | Clade III? | 188 |
| *B. ferriligni* | CCBAU 51502^T^ | - | *Erythrophleum fordii* | China | Pantropical | *B. elkanii* | Clade III (III.3) | 188 |
| *B. forestalis* | INPA54B^T^ | + | Amazon legume tree | Brazil | Pantropical | *B. japonicum* | Clade III (III.3) | 189 |
| *B. ganzhouense* | CCBAU 101088^T^ RITF806T | - | *Acacia melanoxylon* | China | Australia | *B. japonicum* | Clade I | 190 |
| *B. guangdongense* | CCBAU 51649^T^ | - | *Arachis hypogaea* | China | Eastern Asia? | *B. japonicum* | A new, distant clade? | 191 |
| *B. guangxiense* | CCBAU 53363^T^ | - | *Arachis hypogaea* | China | Pantropical | *B. japonicum* | Clade III (III.3) | 191 |
| *B. huanghuaihaiense* | CCBAU 23303^T^ | 1 | *Glycine max* | China | Pantropical | *B. japonicum* | Clade III (III.3) | 192 |
| *B.* *icense* | LMTR 13^T^ | 1 | *Phaseolus lunatus* | Peru | Pantropical | *B. elkanii* | Clade IV | 193 |
| *B. ingae* | BR 10250^T^ | - | *Inga laurina* | Brazil, Roraima | N.-S. America South-east Asia | *B. japonicum* | Clade VII | 144 |
| *B. iriomotense* | EK05^T^ | 1 | *Entada koshunensis* | Japan, Okinawa | N.-S. America South-east Asia | *B. japonicum* | Clade VII | 143 |
| *B. japonicum* | USDA 6^T^ | 1 | *Glycine max* | Japan | Pantropical | *B. japonicum* | Clade III (III.3) | 112 |
| *B. jicamae* | PAC68^T^ | 1 | *Pachyrhizus erosus* | Honduras | Temperate-tropical N. America | *B. elkanii* | Clade XVI | 149 |
| *B. kavangense* | 14-3^T^ | - | *Vigna unguiculata* | Namibia, Kavango | Pantropical | *B. japonicum* | Clade III (III.3) | 194 |
| *B. lablabi* | CCBAU 23086^T^ | 1 | *Lablab purpureus* | China | Pantropical | *B. elkanii* | Clade IV | 195 |
| *B. liaoningense* | 2281^T^ | - | *Glycine max* | China | Pantropical | *B. japonicum* | Clade III (III.3) | 196 |
| *B. lupini* | USDA 3051^T^ | - | *Lupinus angustifolius* | USA | Mediterranean, Europe, temperate N.-S. America | *B. japonicum* | Clade II | 69 |
| *B. manausense* | BR 3351^T^ | 1 | *Vigna unguiculata* | Brazil | N.-S. America. South-east Asia | *B. japonicum* | Clade VII | 145 |
| *B. macuxiense* | BR 10303^T^ | 1 | *Centrolobium paraense* | Brazil | N.-S. America | *B. elkanii* | Clade III (III.4) | 182 |
| *B. mercantei* | SEMIA 6399^T^ | 1 | *Deguelia costata* | Brazil | N.-S. America | *B. elkanii* | Clade III (III.4) | 197 |
| *B. namibiense* | 5-10^T^ | - | *Lablab purpureus* | Namibia | Pantropical | *B. elkanii* | Clade IV | 142 |
| *B. neotropicale* | BR 10247^T^ | 1 | *Centrolobium paraense* | Brazil, Roraima | Tropical S. America | *B. japonicum* | Clade XIX | 198 |
| *B. oligotrophicum* | S58^T^ | 1 | *-* | - | - | *B. japonicum* | Non-nodulating | 199 |
| *B. ottawaense* | OO99^T^ | 1 | *Glycine max* | Canada | Pantropical | *B. japonicum* | Clade III (III.3) | 200 |
| *B. pachyrhizi* | PAC48^T^ | 2 | *Pachyrhizus erosus* | Honduras | Pantropical | *B. elkanii* | Clade III (III.3) | 149 |
| *B. paxllaeri* | LMTR 21^T^ | - | *Phaseolus lunatus* | Peru | Pantropical | *B. elkanii* | Clade IV | 193 |
| *B. retamae* | Ro19^T^ | 1 | *Retama sphaerocarpa* | Morocco | Pantropical | *B. elkanii* | Clade IV | 88 |
| *B. rifense* | CTAW71^T^ | - | *Cytisus villosus* | Morocco | Mediterranean, Europe, temperate N.-S. America | *B. japonicum* | Clade II | 52 |
| *B. sacchari* | BR10280^T^ | 1 | *Vigna unguiculata* | Brazil | Tropical S. America | *B. japonicum* | Clade XII | 201 |
| *B. shewense* | ERR11^T^ | 1 | *Erythrina brucei* | Ethiopia | Pantropical | *B. japonicum* | Clade III (III.3) | 202 |
| *B. stylosanthis* | BR 446^T^ | 1 | *Stylosanthes guianensis* | Brazil | N.-S. America South-east Asia | *B. japonicum* | Clade VII | 146 |
| *B. subterraneum* | 58 2-1^T^ | - | *Vigna subterranea* | Namibia, Kavango | Pantropical | *B. japonicum* | Clade III (III.3) | 203 |
| *B. tropiciagri* | SEMIA6148^T^, CNPSo 1112^T^ | 1 | *Neonotonia wightii* | Brazil | Pantropical | *B. elkanii* | Clade III (III.3) | 187 |
| *B. valentinum* | LmjM3^T^ | 1 | *Lupinus mariae-josephae* | Spain | Pantropical | *B. elkanii* | Clade IV | 76 |
| *B. vignae* | 7-2^T^ | - | *Vigna unguiculata* | Namibia, Kavango | Pantropical | *B. japonicum* | Clade III (III.3) | 204 |
| *B. viridifuturi* | SEMIA 690^T^ | 1 | *Centrosema pubescens* | Brazil | Pantropical | *B. elkanii* | Clade III (III.3) | 205 |
| *B. yuanmingense* | CCBAU 10071^T^ | 1 | *Lespedeza cuneata* | China | Pantropical | *B. japonicum* | Clade III (III.3) | 206 |

**Table S2.** The list of Legume genera belonging to *nifD* gene Clades that comprise Genisteae *Bradyrhizobium* symbionts.

| **Clade** | **Legume Genera** | **Origin** |
| --- | --- | --- |
| **Clade II** | *Acmispon* | USA |
|  | *Cytisus* | Europe |
|  | *Genista* | Europe |
|  | *Laburnum* | Europe |
|  | *Lotus* | Europe; USA; Mexico |
|  | *Lupinus* | Europe; USA; Peru; Bolivia; Ecuador |
|  | *Ornithopus* | Europe |
|  | *Ulex* | Europe |
| **Clade III.3C** | *Abrus* | Australia |
|  | *Albizia* | Ethiopia |
|  | *Arachis* | China |
|  | *Argyrolobium* | South Africa |
|  | *Canavalia* | Costa Rica; USA: Puerto Rico |
|  | *Centrosema* | USA: Texas, North Carolina |
|  | *Chamaecrista* | USA: Puerto Rico, Texas, North Carolina; Australia |
|  | *Crotalaria* | Australia |
|  | *Desmodium* | USA; Australia |
|  | *Faidherbia* | Ethiopia |
|  | *Galactia* | Australia |
|  | *Glycine* | China; Nepal |
|  | *Indigofera* | France: Guadeloupe; Australia |
|  | *Lespedeza* | China; USA: Connecticut |
|  | *Lupinus* | USA: New York |
|  | *Macroptilium* | USA: Puerto Rico; Mexico |
|  | *Macrotyloma* | Zimbabwe |
|  | *Millettia* | Australia |
|  | *Phaseolus* | Mexico; China |
|  | *Rhynchosia* | South Africa |
|  | *sugarcane roots* | Brazil |
|  | *Tephrosia* | Mexico |
|  | *Vigna* | Japan; Nepal; Brazil; China |
| **Clade III.3G** | *Cytisus* | USA |
|  | *Retama* | Spain |
| **Clade III.3B** | *Acacia* | Australia; Portugal |
|  | *Alysicarpus* | Australia |
|  | *Cytisus* | Portugal |
|  | *Galactia* | Australia |
|  | *Retama* | Spain |
|  | *Tephrosia* | Australia |
|  | *Vigna* | Australia |
| **Clade III.2** | *Canavalia* | Mexico; France: Guadeloupe |
|  | *Centrosema* | France: Guadeloupe |
|  | *Chamaecrista* | USA: Puerto Rico, Texas; Mexico |
|  | *Coursetia* | Mexico |
|  | *Crotalaria* | Mexico |
|  | *Desmodium* | Mexico; USA: New York |
|  | *Indigofera* | Mexico |
|  | *Lotus* | Mexico |
|  | *Lupinus* | Brazil |
|  | *Macroptilium* | France: Guadeloupe; Mexico |
|  | *Phaseolus* | Mexico |
|  | *Rhynchosia* | Mexico |
|  | *Zornia* | Mexico |
| **Clade III.4** | *Astragalus* | Mexico |
|  | *Centrolobium* | Brazil |
|  | *Centrosema* | Costa Rica |
|  | *Clitoria* | Panama |
|  | *Crotalaria* | Mexico |
|  | *Dalbergia* | Costa Rica |
|  | *Deguelia* | Brazil |
|  | *Desmodium* | Mexico; USA |
|  | *Dimorphandra* | Brazil |
|  | *Galactia* | USA: North Carolina; Mexico |
|  | *Lonchocarpus* | Panama; France: Guadeloupe |
|  | *Lotus* | Mexico |
|  | *Lupinus* | Brazil; Mexico |
|  | *Machaerium* | Mexico |
|  | *Phaseolus* | Mexico |
|  | *Platymiscium* | Costa Rica |
| **Clade IV** | *Abrus* | Australia |
|  | *Acacia* | Australia |
|  | *Alysicarpus* | Australia |
|  | *Andira* | USA: Puerto Rico |
|  | *Argyrolobium* | South Africa |
|  | *Baptisia* | USA: Texas |
|  | *Canavalia* | France: Guadeloupe; USA: Puerto Rico |
|  | *Chamaecrista* | France: Guadeloupe |
|  | *Crotalaria* | Australia |
|  | *Galactia* | Australia; USA: Texas |
|  | *Genista* | Croatia |
|  | *Indigofera* | Australia; USA: Texas |
|  | *Lablab* | China |
|  | *Laburnum* | Croatia |
|  | *Leobordea* | South Africa |
|  | *Lotononis* | South Africa |
|  | *Lupinus* | Spain |
|  | *Pearsonia* | South Africa |
|  | *Phaseolus* | Peru |
|  | *Retama* | Morocco; Spain |
|  | *Rhynchosia* | USA: Texas |
|  | *Spartium* | Croatia |
|  | *Stylosanthes* | France: Guadeloupe |
|  | *Vigna* | Australia |
| **Clade VII** | *Aeschynomene* | Thailand |
|  | *Andira* | USA: Puerto Rico |
|  | *Centrosema* | Mexico; Costa Rica |
|  | *Clitoria* | USA: North Carolina |
|  | *Cojoba* | Costa Rica |
|  | *Dalbergia* | Costa Rica; USA: Puerto Rico; Panama |
|  | *Desmodium* | France: Guadeloupe; Australia; Panama; Costa Rica |
|  | *Dimorphandra* | Brazil |
|  | *Dioclea* | Panama |
|  | *Galactia* | USA: North Carolina |
|  | *Inga* | France: Guadeloupe; Mexico; Panama; French Guiana; Costa Rica |
|  | *Lonchocarpus* | France: Guadeloupe; Panama; Costa Rica |
|  | *Lupinus* | Brazil |
|  | *Machaerium* | Panama; Mexico; Costa Rica |
|  | *Mucuna* | Costa Rica |
|  | *Ormosia* | France: Guadeloupe |
|  | *Pentaclethra* | Costa Rica |
|  | *Piscidia* | France: Guadeloupe |
|  | *Platymiscium* | Costa Rica |
|  | *Platypodium* | Panama |
|  | *Pseudosamanea* | Costa Rica |
|  | *Pterocarpus* | Philippines |
|  | *Rhynchosia* | Panama |
|  | *Stylosanthes* | Brazil |
|  | *Swartzia* | France: Guadeloupe |
|  | *Vigna* | Panama; Brazil |
|  | *Wallaceodendron* | Philippines |
|  | *Zygia* | French Guiana |
| **Clade XVI** | *Andira* | Panama |
|  | *Dalbergia* | France: Guadeloupe |
|  | *Lotus* | USA: California |
|  | *Lupinus* | USA: Washington State |
|  | *Pachyrhizus* | Honduras |
